# Supplementary material for: A systematic review of exergame usability as home-based balance training tool for older adults usability of exergames as home-based balance training
Source: PLoS One. 2024 Aug 22;19(8):e0306816. doi: 10.1371/journal.pone.0306816 (PMC11340971; doi:10.1371/journal.pone.0306816)
Supplement: S2 File — (DOCX) [file pone.0306816.s002.docx]

**Search strategies for all databases**

No filters will be used.

| **DATABASE** | **Search Terms** |
| --- | --- |
| **MEDLINE (Pubmed)** | ("aged"[MeSH] OR "elderly"[tiab] OR "older adult"[tiab] OR "older adults"[tiab] OR "elder"[tiab] OR "senior"[tiab] OR "elders"[tiab] OR "seniors"[tiab] OR "geriatrics"[MeSH]) AND ("exergaming"[MeSH] OR "exergame"[tiab] OR "exergames"[tiab] OR "virtual reality exercise"[tiab] OR "virtual reality exercises"[tiab] OR "video games"[MeSH] OR "video games"[MeSH] OR "active video gaming"[tiab] OR "active video gaming"[tiab] OR "nintendo wii"[tiab] OR "wii fit"[tiab] OR "xbox"[tiab] OR "kinect"[tiab] OR "kinect-based"[tiab]) AND ("user-centered design"[MeSH] OR "user centered design"[tiab] OR "usability"[tiab] OR "usability testing"[tiab] OR "postural balance"[MeSH] OR "postural control"[tiab] OR "postural controls"[tiab] OR "posture equilibrium"[tiab] OR "postural equilibrium"[tiab] OR "balance"[tiab] OR "body balance"[tiab] OR "postural stability"[tiab]) |
| **EMBASE (Elsevier)** | ('aged'/exp OR aged OR 'elderly'/exp OR elderly OR 'older adult'/exp OR 'older adult' OR 'older adults'/exp OR 'older adults' OR elder OR elders OR senior OR seniors OR 'geriatric'/exp OR geriatric OR 'geriatrics'/exp OR geriatrics) AND (exergames OR exergame OR exergaming OR 'virtual reality exercise' OR 'virtual reality exercises' OR 'active-video gaming' OR 'active video gaming' OR 'video game' OR 'video games' OR 'nintendo wii' OR 'wii fit' OR xbox OR kinect OR 'kinect-based') AND ('user centered design' OR 'user-centered design' OR 'usability' OR 'usability testing' OR 'postural balance' OR 'postural control' OR 'postural controls' OR 'posture equilibrium' OR 'postural equilibrium' OR 'balance' OR 'body balance' OR 'postural stability') |
| **Web of Science** | (((ALL=((aged OR elderly OR “older adult” OR “older adults” OR elder OR elders OR senior OR seniors OR geriatric OR geriatrics))) AND ALL=((exergames OR exergame OR exergaming OR “virtual reality exercise” OR “virtual reality exercises” OR “active-video gaming” OR “active video gaming” OR “video game” OR “video games” OR “nintendo wii” OR“wii fit” OR xbox OR kinect OR “kinect-based”) )) AND ALL=((user centered design' OR 'user-centered design' OR 'usability' OR 'usability testing'))) AND ALL=(('postural balance' OR 'postural control' OR 'postural controls' OR 'posture equilibrium' OR 'postural equilibrium' OR 'balance' OR 'body balance' OR 'postural stability')) |
| **Scopus (Elsevier)** | ( aged OR elderly OR "older adult" OR "older adults" OR elder OR elders OR senior OR seniors OR geriatric OR geriatrics ) AND ( exergames OR exergame OR exergaming OR "virtual reality exercise" OR "virtual reality exercises" OR "active-video gaming" OR "active video gaming" OR "video game" OR "video games" OR "nintendo wii" OR "wii fit" OR xbox OR kinect OR "kinect-based" ) AND ( "user centered design" OR "user-centered design" OR "usability" OR "usability testing") AND ( "postural balance" OR "postural control" OR "postural controls" OR "posture equilibrium" OR "postural equilibrium" OR  "balance" OR "body balance" OR "postural stability" ) AND ( "randomized controlled trial" AND "controlled clinical trial" AND randomized AND trial ) |
| **Science Direct** | 1) (elderly OR “older adults” OR seniors)  AND (exergaming OR “virtual reality exercise” OR “active-video gaming” OR “video games”) AND (“user-centered design” OR "postural balance")  2) (aging OR “older adults” OR geriatrics)  AND (exergaming OR "nintendo wii" OR “active-video gaming” OR “xbox”) AND (“usability” OR "postural control")  3) (elderly OR “older adults”)  AND (exergames OR exergaming OR “active-video gaming” OR “video games”) AND (“usability testing” OR "postural balance" OR "postural stability")  4) (aged OR “older adults”)  AND (exergaming OR “active-video gaming” OR “video games”) AND ("user centered design" OR “usability” OR "postural balance" OR "postural stability") |
| **Cochane (CENTRAL)** | *("aged" OR "elderly" OR “older adult” OR “older adults” OR "elder" OR "elders" OR "senior" OR "seniors" OR "geriatric" OR "geriatrics") in Title Abstract Keyword AND ("exergames" OR "exergame" OR "exergaming" OR “virtual reality exercise” OR “virtual reality exercises” OR “active-video gaming” OR “active video gaming” OR “video game” OR “video games” OR “nintendo wii” OR“wii fit” OR "xbox" OR "kinect" OR “kinect-based”) in Title Abstract Keyword AND (“user centered design” OR “user-centered design” OR “usability” OR “usability testing”) in Title Abstract Keyword AND (“postural balance” OR “postural control” OR “postural controls” OR “posture equilibrium” OR “postural equilibrium” OR “posture balance” OR “balance” OR “body balance” OR “postural stability”) in Title Abstract Keyword |
