# Supplementary material for: A systematic review of exergame usability as home-based balance training tool for older adults usability of exergames as home-based balance training
Source: PLoS One. 2024 Aug 22;19(8):e0306816. doi: 10.1371/journal.pone.0306816 (PMC11340971; doi:10.1371/journal.pone.0306816)
Supplement: Attachment — Submitted filename: Response to Reviewers.docx [file pone.0306816.s006.docx]

**RESPONSE TO REVIEWERS**

Response from Medeiros et al.

We are grateful for all comments and suggestions raised by Reviewer 2.

# Journal Requirements

Please review your reference list to ensure that it is complete and correct. If you have cited papers that have been retracted, please include the rationale for doing so in the manuscript text, or remove these references and replace them with relevant current references. Any changes to the reference list should be mentioned in the rebuttal letter that accompanies your revised manuscript. If you need to cite a retracted article, indicate the article’s retracted status in the References list and also include a citation and full reference for the retraction notice.

Response from Medeiros et al.

As recommended, we have reviewed the reference list to ensure it is correct. Nine articles were added to the manuscript. Most of the studies added were to complement the introduction, and one study was added to add to enhance the description of usability. The articles added to the manuscript are:

(4) Yeşilyaprak SS, Yildirim MŞ, Tomruk M, Ertekin Ö, Algun ZC. Comparison of the effects of virtual reality-based balance exercises and conventional exercises on balance and fall risk in older adults living in nursing homes in Turkey. Physiother Theory Pract. 2016;32(3):191–201.

(5) Horak F. Postural orientation and equilibrium: what do we need to know about neural control of balance to prevent falls? Age Aging. 2006;35(2):7–11.

(6) Sedaghati P, Goudarzian M, Ahmadabadi S, Tabatabai-Asl SM. The impact of a multicomponent-functional training with postural correction on functional balance in the elderly with a history of falling. J Exp Orthop. 2022;9(1).

(7) Alshahrani MS, Reddy RS. Kinesiophobia, limits of stability, and functional balance assessment in geriatric patients with chronic low back pain and osteoporosis: a comprehensive study. Front Neurol. 2024; 15:1354444.

(8) Dunsky A. The Effect of Balance and Coordination Exercises on Quality of Life in Older Adults: A Mini-Review. Front Aging Neurosci. 2019;11(318):1–10.

(10) Rose T, Nam CS, Chen KB. Immersion of virtual reality for rehabilitation - Review. Vol. 69, Applied Ergonomics. Elsevier Ltd; 2018. p. 153–61.

(19) Aclan R, George S, Block H, Lane R, Laver K. Middle aged and older adults’ perspectives of their own home environment: a review of qualitative studies and meta-synthesis. BMC Geriatr. 2023;23(707):1–12.

(20) Meghani NAA, Hudson J, Stratton G, Mullins J. Older adults’ perspectives on physical activity and sedentary behavior within their home using socio-ecological model. PLoS One. 2023;18(11):1–24.

(38) Brooke J. SUS - A quick and dirty usability scale. Usability Eval Ind. 1996;189–94.

# Reviewer #1

(No Response)

# Reviewer #2

A) re- #4. The link to the supporting data did not work- so this might be a problem on my end.

Response from Medeiros et al.

Thank you for this comment. We will attach all supporting information files individually and in a compressed folder (zipe file).

B) Overall previous edits were addressed effectively. With the intent of helping you make this paper the best it can me, I found two things:

1) 104- A repeat of line 97, and confusing: at first I thought this was an incomplete sentence, details as a noun instead of a verb, and used being the operative verb here.

2) 330-332. sorry if I did not catch this last time, this statement needs clarification: 310. “the Five times sit-to-stand test and found that the control group showed significantly a lower performance compared exergame group that reduced the time of the test in the post intervention. “In a timed test, a lower score = faster, so this means the control group improved, not the intervention group.

Response from Medeiros et al.

The authors would like to thank you for providing such detailed comments, which are very constructive and will help to improve the manuscript.

1) Thank you for pointing this out. As line 104 was confusing (“The protocol details the complete search strategy used.”), we have chose to remove this excerpt from the manuscript.

2) In fact, we intended to indicate that the reduction in test time post-intervention belonged to the experimental group. We acknowledge that the excerpt highlighted by the reviewer was confusing.

\# Study data (Campo-Prieto P, Cancela-Carral JM, Rodríguez-Fuentes G. Feasibility and Effects of an Immersive Virtual Reality Exergame Program on Physical Functions in Institutionalized Older Adults: A Randomized Clinical Trial. Sensors. 2022;22(6742):1–15):

- Experimental group (seconds) = Pre (15.56±4.52), Post (13.81±3.46), Variation (-1.75).
- Control group (seconds) = Pre (21.19±12.63), Post (25.57±14.15), Variation (+4.38)* p<0.005.
- The study by Campo-Prieto, Cancela-Carral and Rodrígues-Fuentes (2022) showed that the control group exhibited a significantly worsening compared to the experimental group.

Therefore, we have adjusted the text and included the performance variation for each group to carify the information.
